# Public attitudes in the United States toward insurance coverage for in vitro fertilization and the provision of infertility services to lower income patients

**DOI:** 10.1016/j.xfre.2021.09.002

**Published:** 2021-09-20

**Authors:** Jacqueline R. Ho, Lusine Aghajanova, Evelyn Mok-Lin, Jacquelyn R. Hoffman, James F. Smith, Christopher N. Herndon

**Affiliations:** a Division of Reproductive Endocrinology and Infertility, Department of Obstetrics & Gynecology, University of Southern California, Los Angeles, California; b Division of Reproductive Endocrinology and Infertility, Department of Obstetrics & Gynecology, Stanford University, Palo Alto, California; c Division of Reproductive Endocrinology and Infertility, Department of Obstetrics, Gynecology, & Reproductive Sciences, University of California, San Francisco, California; d University of Arizona School of Medicine, Tucson, Arizona; e Department of Urology, University of California, San Francisco, California; f Philip R. Lee Institute for Health Policy Studies, University of California, San Francisco, California; g Division of Reproductive Endocrinology and Infertility, Department of Obstetrics & Gynecology, University of Washington, Seattle

**Keywords:** Access to care, infertility, insurance, IVF, public opinion

## Abstract

**Objective:**

To assess attitudes and factors that influence public opinion in the general US population toward insurance coverage and provision of infertility care to lower income patients.

**Design:**

Cross-sectional survey.

**Setting:**

Online.

**Patient(s):**

A nationally representative sample of US residents.

**Intervention(s):**

Questionnaire with multiple choice and open response questions.

**Main Outcome Measure(s):**

Public attitudes toward in vitro fertilization and infertility care coverage for lower income patients.

**Result(s):**

A total of 1,027 (90.2%) participants completed the survey, among whom 620 (60.4%) had private insurance, 275 (26.8%) had Medicare/Medicaid, and 56 (5.5%) were uninsured. The majority (916, 89.2%) did not consider infertility a disease. Over half of the respondents (568, 55.3%) supported private insurance coverage of infertility services, including for in vitro fertilization. Most respondents, 735 (71.6%) believed that the prevalence and psychosocial impact of infertility were equal among the lower and higher income people. The majority of respondents with an opinion (512, 67.6%) believed that doctors should provide infertility treatments regardless of the income level of the patients. Of supporters, 40.1% believed in the right to have a family regardless of income, and 38.2% believed that doctors had a social responsibility to provide infertility services. After adjusting for covariates, age <45 years, noncollege graduates, desiring more children, believing that infertility was a disease, and residence in the Northeast region remained significant predictors for support of private insurance coverage.

**Conclusion(s):**

Public perception of infertility as a disease is one of the strongest predictors of support for insurance coverage for infertility services, underscoring the need for enhanced advocacy and education in the general public.

**Discuss:** You can discuss this article with its authors and other readers at https://www.fertstertdialog.com/posts/xfre-d-21-00112

In the United States, approximately 1 in 8 couples are affected by infertility, yet only a fraction are able to access care (1, 2). There are many barriers that limit access to care in the United States, of which the greatest is the cost of health care (2). Currently, 19 states have laws mandating some insurance coverage for infertility treatment, of which only 8 specify that in vitro fertilization (IVF) must be covered by insurance in some capacity, and even fewer mandating comprehensive coverage (3, 4, 5). Few published studies to date have assessed perceptions of the general public on insurance coverage of IVF (6, 7). These studies, which primarily involve cohorts in Europe and Australia, demonstrate an overall positive support for insurance coverage (6, 7). No recent study has examined public attitudes toward coverage of infertility services in the United States.

Entirely unexamined to our knowledge are the opinions of the public in the United States on the support and provision of infertility care to underserved communities, which include immigrants who may lack legal resident status. Public-funded or subsidized health care for immigrants without legal residency remains a highly charged political issue (8). As of 2015, an estimated 43.3 million immigrants reside in the United States (9). In addition to financial challenges, immigrants face substantial and sometimes insurmountable barriers to accessing health care, including health literacy, language, bureaucracy, legal barriers, racism, and discrimination (10). The high out-of-pocket cost of infertility care in the United States disproportionately impacts individuals from a low resource, largely immigrant communities. Additionally, many may have limited eligibility to qualify for financing or loan programs to help financially access treatments (11).

Policy changes, whether they be state level legislative mandates for insurance coverage for IVF or inclusion of infertility coverage into public health plans, are unlikely to take effect without understanding the opinions and needs of the public. In this study, we sought to assess attitudes and factors that impact public opinion toward insurance coverage and provision of infertility care to lower income patients.

## Materials and methods

### Study Design and Cohort

This study was an online-based cross-sectional survey of a representative sample of US residents using SurveyMonkey Audience, a professional online platform with >30 million volunteer participants. Prior studies have used this platform to examine national public opinion on reproductive health topics (12, 13). SurveyMonkey Audience recruited US residents aged 18–75 years to approximate census data on the basis of distributions of age and sex. Age and sex were self-reported. Inclusion in this study required participants to be literate in English and to have access to a computer and internet. Investigators also had options to purchase a number of responses and targeted demographics. Although direct financial compensation for every respondent was not offered, in exchange for their participation, SurveyMonkey Audience respondents were offered by the company to receive a $0.50 donation to a charity of their choice for each survey they completed or entry into a weekly sweepstake to win $100. The introduction page provided basic background information and definitions of infertility, in vitro fertilization, and the average treatment costs. Surveys were sent to 4 equally sized groups: men aged 18–44 years, men aged 45–75 years, women aged 18–44 years, and women aged 45–75 years. Demographic and socioeconomic characteristics were provided by SurveyMonkey or were obtained from the survey questions (Appendix 1, available online). Personal identifiers and protected health information were not collected. Respondents were from 49 US states (all but North Dakota), and a demographic breakdown of the respondents alongside US Census Data can be found in Supplemental Table 3 (available online) (14). This study was exempted by the institutional review board of the University of California, San Francisco. The survey was distributed in April 2017.

### Questionnaire

Before beginning the survey, the respondents were asked to read a summary page introducing the purpose and content of the survey, at which time they elect to decline to participate. The questionnaire was started with 10 questions about demographics and socioeconomic information (Appendix 1, available online). We gathered information about reproductive characteristics, including a personal history of infertility, knowing someone with infertility, parity, and plans for future childbearing. This was followed by 11 questions regarding the definition of infertility and attitudes regarding insurance coverage of infertility services, including IVF, to a lower income population. We also assessed the reasons for support or nonsupport toward the coverage of infertility services. For these questions, participants were allowed to check >1 response and “other,” in which they entered a response by free text. In our last question, we presented 4 scenarios of patients with different medical and social indications for IVF and asked participants to choose for whom IVF should be covered (Supplemental Table 2, available online). Answers were in multiple-choice format, with the option for a single response. For questions inquiring reasons for support or nonsupport, participants were given responses with the option to choose >1 answer. These questions also had an “other” response option, with the ability for participants to write in responses. Freehand responses were either recategorized by the primary investigator into existing answer categories if the response was similar to the answer choice or otherwise classified into an “other” group. At the end of our survey, we posed 4 potential scenarios for couples requiring IVF to build families. We stated that the woman was 30 years old and married in a heterosexual relationship for all case examples. The couple had a combined income of $31,020, representing 200% above the federal poverty line for a 2-person household in the United States in 2016, a threshold commonly used as a financial eligibility criterion by assistance programs (15). In addition, we reported that the average expense for raising a child in a 2-child married-couple family was $9,500/year (16).

### Statistical Analysis

Descriptive statistics, including age group, sex, ethnicity, religion, education level, partner status, insurance status, and geographic location in the United States, are presented in numbers (percentages). Logistic regression was used to test associations between support, nonsupport, or neutral status with each demographic category grouping (age, sex, college status, income bracket, partner status, race/ethnicity, religion, insurance status, region, history of infertility, knowing someone with infertility, desiring children, and parental status). *P* values listed are for the comparisons tested (based on each predictor variable category, i.e., age <45 vs. ≥45 years). Unadjusted and adjusted risk ratios were calculated using multinomial logistic regression to compare demographic and socioeconomic characteristics of those that supported, did not support, or were neutral toward insurance coverage of infertility services. Predictor variables were classified as categorical, and the reference groups were defined as the largest groups. Covariates for a multivariable model were chosen a priori or had significant associations with bivariate analysis using chi-square, and included age, sex, partner status, education level, income, and knowledge of someone with infertility, thoughts about future childbearing, the definition of infertility as a disease, religious status, and the US region. The following are referent groups for the multivariable analysis: age group (<45 years old), sex (female), partner status (unpartnered), education status (not a college graduate), income (<$100,000), knowing someone with infertility (no), desire more children (no), believe infertility is a disease (no), religion (atheist/agnostic), and the US region (Northeast region). We performed tests for linear trend using chi-square analysis for variables including age (age category), household income, and educational status. The 95% confidence intervals were provided, and 2-tailed tests were used for *P* values, with significance defined as *P*<.05. Statistical software used was Stata (version 15.1, College Station, TX).

## Results

### Demographics

A total of 1,027 of 1,138 (90.2%) participants completed the survey. Of the 1,138 (9.8%) respondents, 111 were excluded for not completing essential questions in the survey (questions 1–10). The demographics (Tables 1 and 2) of the participants were balanced and mostly reflective of the national statistics. Two hundred and sixteen (21%) respondents were in the age group 18–29 years, 269 (26.2%) in the age group 30–44 years, 281 (27.4%) in the age group 45–59 years, and 261 (25.4%) in the age group ≥60 years.

**Table 1:** Demographic and socioeconomic status characteristics stratified by support and nonsupport of in vitro fertilization coverage.

Demographics	Support insurance coverage (n = 568)	Do not support insurance coverage (n = 189)	Neutral (n = 270)	*P* value
Age <45 y (n = 485)	303 (62.5%)	69 (14.2%)	113 (23.3%)	
Age ≥45 y (n = 542)	265 (48.9%)	120 (22.1%)	157 (29%)	<.001
Female (n = 535)	314 (58.7%)	98 (18.3%)	123 (22.3%)	
Male (n = 492)	254 (51.6%)	91 (18.5%)	147 (29.9%)	.03
Not college graduate (n = 405)	229 (56.5%)	59 (39.3%)	117 (28.9%)	
College graduate (n = 622)	339 (54.5%)	130 (20.9%)	153 (26.2%)	.027
Income <$100,000 (n = 647)	370 (57.2%)	102 (15.8%)	175 (27%)	
Income ≥$100,000 (n = 380)	198 (52.1%)	87 (22.9%)	95 (25%)	.03
Partnered (n = 658)	375 (57%)	121 (18.4%)	162 (24.6%)	
Unpartnered (n = 369)	193 (52.3%)	68 (18.4%)	108 (29.3%)	.24
Race/ethnicity				
White/European (n = 810)	431 (65.5%)	160 (19.8%)	219 (27%)	
Hispanic/Latino (n = 59)	45 (76.3%)	4 (6.8%)	10 (16.9%)	
Black/African American (n = 67)	41 (61.2%)	8 (11.9%)	18 (26.9%)	
Asian/Pacific Islander (n = 36)	23 (63.9%)	4 (11.1%)	9 (25%)	
Native American (n = 8)	5 (62.5%)	2 (25%)	1 (12.5%)	
Other/multiple (n = 47)	23 (48.9%)	11 (23.4%)	13 (27.7%)	.06
Religion				
Protestant Christian (n = 317)	160 (50.5%)	66 (20.8%)	91 (28.7%)	
Catholic Christian (n = 234)	132 (56.4%)	43 (18.4%)	59 (25.2%)	
Jewish (n = 27)	14 (51.9%)	3 (11.1%)	10 (37%)	
Muslim (n = 19)	11 (57.9%)	0 (0%)	8 (42.1%)	
Buddhist (n = 11)	4 (36.4%)	0 (0%)	7 (63.6%)	
Hindu (n = 4)	1 (25%)	1 (25%)	2 (50%)	
Atheist/agnostic (n = 193)	110 (57%)	45 (23.3%)	38 (19.7%)	
Other (n = 222)	136 (61.3%)	31 (14%)	55 (24.7%)	.006
Insurance status				
Uninsured (n = 56)	30 (53.6%)	14 (25%)	12 (21.4%)	
Private (n = 620)	355 (57.3%)	113 (18.2%)	152 (24.5%)	
Medicare (n = 206)	95 (46.1%)	46 (22.3%)	65 (31.6%)	
Medicaid (n = 69)	45 (65.2%)	4 (5.8%)	20 (29%)	
Other (n = 76)	43 (56.6%)	12 (15.8%)	21 (27.6%)	.37
US region				
Northeast (n = 207)	135 (65.2%)	25 (12.1%)	47 (22.7%)	
Midwest (n = 233)	125 (53.6%)	49 (21%)	59 (25.3%)	
South (n = 317)	173 (54.6%)	60 (18.9%)	84 (26.5%)	
West (n = 255)	124 (48.6%)	55 (21.6%)	76 (29.8%)	.26

**Table 2:** Characteristics of respondents with respect to infertility and personal reproductive goals.

Reproductive characteristics	Insurance coverage	No insurance coverage	Neutral	*P* value
No infertility (n = 904)	496 (54.9%)	168 (18.6%)	240 (26.5%)	
History of infertility (n = 123)	72 (58.5%)	27 (22%)	30 (24.4%)	.75
Does not know someone with infertility (n = 461)	231 (50.1%)	88 (19.1%)	142 (30.8%)	
Knows someone with infertility (n = 566)	337 (60.6%)	101 (17.8%)	128 (22.6%)	.005
Does not desire a child/children (n = 822)	422 (51.3%)	167 (20.3%)	233 (28.3%)	
Desire a child/children (n = 205)	146 (71.2%)	22 (10.7%)	37 (18%)	<.001
Does not have children (n = 440)	240 (54.5%)	91 (20.7%)	109 (24.8%)	
Has a child/children (n = 587)	328 (55.6%)	98 (16.7%)	161 (27.4%)	.23

Overall, 568 (55.3%) participants supported insurance coverage of infertility and IVF services, 189 (18.4%) participants did not support insurance coverage of infertility and IVF services, and 270 (26.3%) participants were neutral. Tables 1 and 2 and Supplemental Table 1 (available online) show characteristics associated with support vs. nonsupport of insurance coverage. Age <45 years, women, noncollege graduates, an income of <$100,000, and living in the Northeast region were characteristics of those more likely to support insurance coverage (Table 1). We assessed how much people would be willing to pay per month if the insurance covered IVF. Of the 1,027 (42.7%) participants, 439 were not willing to pay extra on premiums for IVF coverage. Meanwhile, 378 (36.8%) participants were willing to pay extra on their monthly premiums for this benefit. Of those willing to pay more, 218 (57.6%) were willing to pay $5 per month, 88 (23.3%) were willing to pay $10 per month, and 72 (19%) were willing to pay $20 per month.

**Figure 1:**
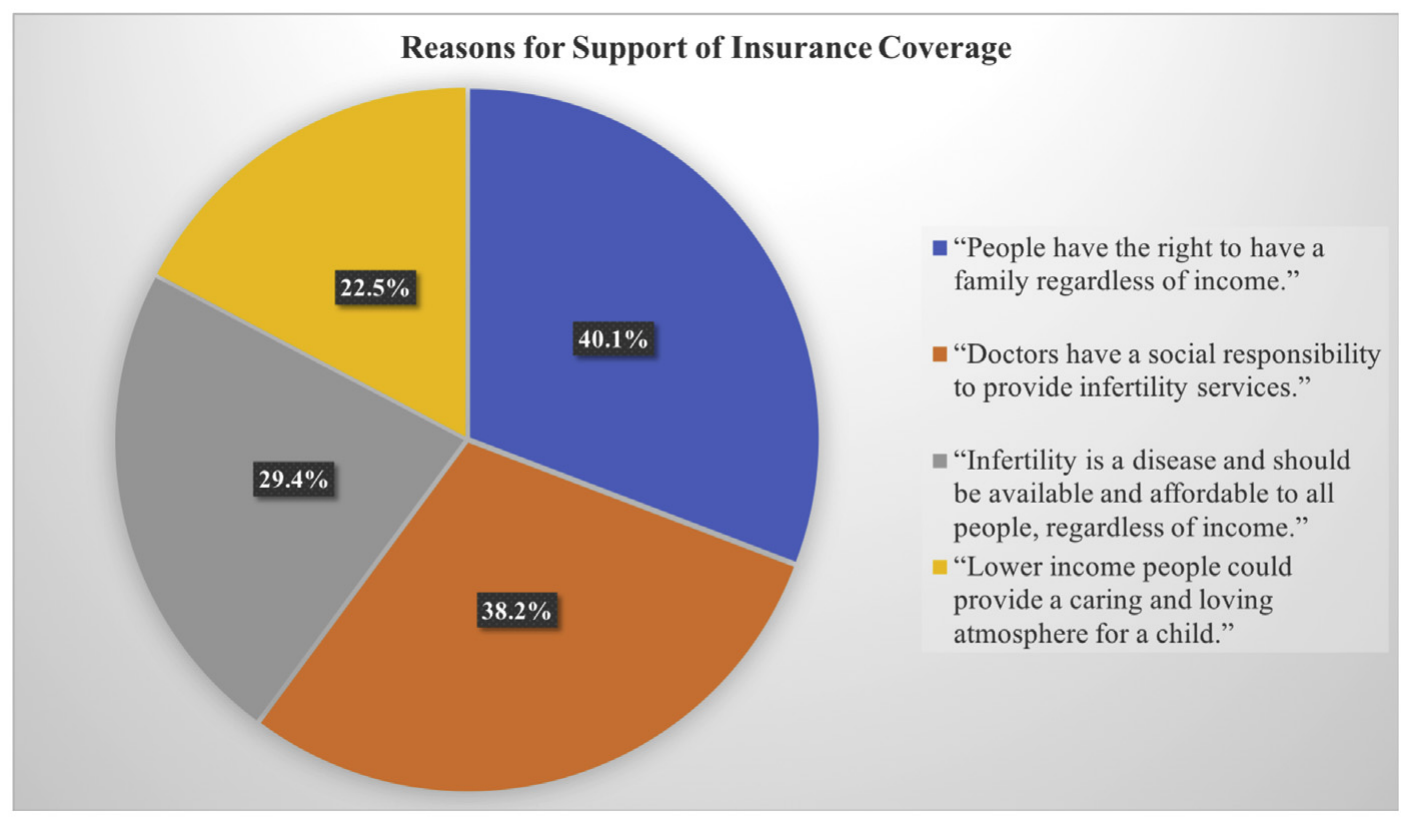
Visual depiction of reasons for support and nonsupport of insurance coverage for infertility services.

We assessed reasons for support of insurance coverage (Figure 1, Table 3). Of those that supported insurance coverage (n = 568), 228 (40.1%) believed that physicians had a social responsibility to provide services, including IVF, to all people regardless of income status; 167 (29.4%) believed that infertility was a disease and that treatment should be available and affordable to everyone, regardless of income; 284 (50%) believed that all infertile people should have the right to have a family, regardless of income; and 137 (24.1%) supporters believed that lower income people could provide a caring and loving atmosphere for a child (Supplemental Table 1).

In a multivariable analysis, age <45 years, noncollege graduates, those desiring more children, and living in the Northeast region remained predictors for supporting the coverage of infertility and IVF services (Table 4).

**Table 3:** Attitudes of respondents on infertility as a disease and societal obligations regarding access to care.

Attitudes	Insurance coverage	No insurance coverage	Neutral	*P* value
Infertility is a disease (n = 286)	222 (77.6%)	16 (5.6%)	48 (16.8%)	
Infertility is not a disease (n = 741)	346 (46.7%)	173 (23.3%)	222 (30%)	<.001
Doctors have social responsibility (n = 217)	172 (79.3%)	15 (6.9%)	30 (13.8%)	
Doctors have no social responsibility (n = 810)	396 (48.9%)	174 (21.5%)	240 (29.6%)	<.001
Immigrants should have access (n = 339)	232 (68.4%)	29 (8.6%)	78 (23%)	
Immigrants should not have access (n = 688)	336 (48.8%)	160 (23.3%)	192 (27.9%)	<.002

**Table 4:** Multivariable analysis of support for insurance coverage of infertility.

Variable	Unadjusted RR	Adjusted RR	*P* value
Age (y)			
≥45	Ref	Ref	
<45	0.5 (0.36–0.71)	0.63 (0.42–0.94)	.03
Sex			
Female	Ref	Ref	
Male	0.87	0.96 (0.67–1.38)	.83
Partner			
No	Ref	Ref	
Yes	1.1 (0.77–1.5)	1.2 (0.83–1.7)	.34
Education			
Not college graduate	Ref	Ref	
College graduate	0.67 (0.47–0.95)	0.62 (0.42–0.91)	.01
Income			
<$100,000	Ref	Ref	
≥$100,000	0.91 (0.76–1.1)	0.86 (0.72–1.02)	.08
Know someone with infertility			
No	Ref	Ref	
Yes	1.27 (0.9–1.8)	1.1 (0.8–1.66)	.45
Desire more children			
No	Ref	Ref	
Yes	1.2 (1.6–4.26)	1.82 (1.1–3.2)	.04
Believe infertility is a disease			
No	Ref	Ref	
Yes	1.93 (1.39–2.5)	6.8 (3.9–11.8)	<.001
Atheist/agnostic			
No	Ref	Ref	
Yes	0.77 (0.52–1.14)	0.68 (0.44–1.0)	.08
Northeast region of the United States			
No	Ref	Ref	
Yes	2.1 (1.3–3.3)	2.4 (1.7–3.9)	<.001

*Note:* Ref = reference; RR = relative risk.

### Attitudes Toward Infertility

When examining attitudes toward infertility, the majority did not believe that infertility was a disease (n = 741, 72.2%). This was a significant predictor for nonsupport of IVF coverage (*P*<.001). Of all respondents, over half (n = 568, 55.3%) supported insurance coverage of infertility services, including IVF; 189 (18.4%) did not think it should be covered; and 270 (26.3%) were neutral. Seven hundred and thirty-five (71.6%) participants believed that the prevalence and psychosocial impact of infertility were equal among lower and higher income people. Although 339 (33%) participants supported access to infertility services by immigrants, 688 (67%) participants did not support physicians providing treatment to illegal immigrants with infertility that might result in a child with rights and benefits of US citizenship. The support of immigrant access was a significant predictor for support of insurance coverage *(P*<.002). Among those who reported support or nonsupport of insurance coverage (n = 757), 67.6% (n = 512) participants believed that doctors should provide infertility treatments regardless of income level. This was a significant predictor for support of insurance coverage (*P*<.002). Of supporters, 40.1% believed in the right to have a family regardless of income, and 38.2% believed that doctors had a social responsibility to provide infertility services (Table 3).

With respect to our 4 scenarios of patients needing IVF, 713 (69.4%) participants supported coverage for a woman with tubal disease; 683 (66.5%) supported coverage for fertility preservation for a woman with recently diagnosed breast cancer and no children; 403 (39.2%) supported coverage for a woman with 2 children and a history of tubal ligation performed nonelectively in her native country, who desired future children; and 280 (27.3%) supported coverage for a woman with 3 children (with a previous partner) and a prior tubal ligation, who desired children with a new partner.

## Discussion

In our study, the majority (55.3%) of the public surveyed supported private insurance coverage of infertility services, including IVF. The strongest predictors for coverage support were as follows: respondent age <45 years old, education level, living in the Northeast region, desiring more children, and considering infertility to be a disease. The belief that infertility is not a disease was one of the biggest predictors among respondents who did not support insurance coverage of infertility services. In comparison, individuals who considered infertility a disease (28%) were almost 7-fold more likely to support coverage. The findings of our study indicate major gaps in the knowledge of the public regarding infertility as a disease, supporting earlier findings in the literature (7). Health organizations have been slow to recognize infertility as a disease publicly. As late as 2009, the World Health Organization defined infertility as a disease, and the American Medical Association followed in 2017 (17, 18). Increased public advocacy and education are necessary to yield policy changes.

A comparative economic analysis of 6 developed countries showed that the cost of an IVF cycle was the highest in the United States, with utilization correspondingly the lowest (19). Because of the cost of health care delivery in the United States, mandated insurance coverage is a requisite intervention for broadly expanding access to care (5, 20, 21). In the absence of health plan coverage, access to care and family building is available only for those who can afford it. Insurance mandated coverage increases utilization by almost 300% (22). Studies of IVF utilization in mandated states show nominal increases in cost proportional to other health care costs, <0.5%–0.85% of overall health care expenditures with nominal increases in premiums (23, 24). Additional benefits conferred with state-mandated insurance coverage of infertility services include a higher percentage of single embryo transfer, which can decrease the rates of higher order multiple pregnancies, preterm birth, and low birth weight (25, 26, 27, 28). Further, studies support the role of state-mandated insurance coverage in improving access to care and utilization for African Americans and other minority groups, although utilization remains underrepresented, indicating the presence of other barriers (29, 30).

The majority of respondents with an opinion (n = 512, 67.6%) believed that doctors should provide infertility treatments regardless of the income level of patients. Of all respondents, 18.4% did not support any insurance coverage of infertility services or IVF. The most commonly cited reasons were that IVF is elective, it is not a social responsibility (n = 112, 59.3%), and that the wellbeing of a child born in a lower income family may be at risk (n = 67, 35.4%). Almost 40% (n = 70, 37%) of respondents who did not support insurance coverage expressed concern that treatment would increase the lower income population and might strain the natural, financial, and social resources. Of all survey respondents, many (445, 43.3%) do not support public health coverage for IVF for lower income patients.

Of the 4 hypothetical scenarios for treatment coverage that we surveyed, respondent support for treatment coverage for patients with lower income was highest for the childless patient with blocked fallopian tubes and childless patient with a cancer diagnosis. It could not be ascertained from responses if support was because their infertility in the circumstances was viewed through the lens of a medical condition or because these patients had no children or both factors. For the patients who had tubal ligations and had children, support was approximately 1.7–2-fold lower for infertility services. Less than one-third (280, 31.2%) supported IVF for a patient, with a prior history of tubal ligation, now with a new partner.

Notably, two-thirds (668, 67%) of all respondents did not support physicians providing treatment to illegal immigrants with infertility that might result in a child with rights and benefits of US citizenship. Although studies of immigrant populations in the United States are limited, the experience and impact of infertility mirrors what is experienced in their countries of origin (31, 32, 33, 34). These patients experience significant untreated disease burdens in their communities, resulting in profound emotional, social, and financial impacts. The World Health Organization has ranked infertility as the fifth leading generator of disability among the population of all women under 60 worldwide (35). Awareness of the prevalence and impact of infertility in these demographics and empathy is further constrained by biases and misconceptions among some that lower income populations have high fertility rates and thus do not need fertility care (10). This can lead to underdiagnosis and be used to condone the lack of attention to infertility in these groups (36). In our survey, 403 (39%) did not support insurance coverage for IVF in a lower income immigrant patient, even in the setting of an unconsented tubal ligation in their prior country. This sobering finding indicates a great need for public education and efforts to address the profound disparities and untreated disease burden in immigrant communities in the United States. Immigrant populations suffer from the convergence of factors that can detract from public empathy and support, including discrimination as targets of political ideology, racism toward those of color, and lack of economic resources, representation, and advocacy. The findings regarding public attitudes toward infertility coverage in lower income patients are unreported in the literature and of importance in the context of the growing interest of professional organizations and health providers in reducing health disparities and expanding access to care. Strengths of the study include the ability to survey a population mostly representative of the diversity of the United States concerning age, sex, race/ethnicity, religious preference, income, education level, and geographic location. Limitations of the study include methodology that required English literacy and access to the computer and internet, thus potentially constraining the generalizability of the findings to the entire US population.

In closing, many in the United States struggling with infertility face considerable barriers in access to care. These barriers are particularly magnified and often insurmountable for those from lower income and immigrant communities. The findings of our study point to the need for public education to change attitudes, help reduce biases, and improve empathy. Improving public support and awareness is critical to advance legislative or health policy change to expand access and to address the substantial untreated disease burden that exists today in our communities. The lack of insurance coverage implies that infertility is a condition undeserving of financial assistance and minimizes both its impact and importance to patients (37). In our study, nearly three-quarters of respondents did not consider infertility to be a disease. Importantly, public support for insurance coverage for infertility services was almost 7-fold higher if infertility is viewed as a disease, highlighting the importance of this messaging in public education and advocacy.

## Conclusion

Public perception of infertility as a disease is one of the strongest predictors of support for insurance coverage for infertility services, underscoring the need for enhanced advocacy and education in the general public.
