# Supplementary material for: Public attitudes in the United States toward insurance coverage for in vitro fertilization and the provision of infertility services to lower income patients
Source: F S Rep. 2021 Sep 20;3(2 Suppl):122–9. doi: 10.1016/j.xfre.2021.09.002 (PMC9349243; doi:10.1016/j.xfre.2021.09.002)
Supplement: Appendix 1 [file mmc1.docx]

**APPENDIX**

**Appendix 1: Survey instrument**

**Public Perceptions of Providing Infertility Services to Low Income Patients**

**Survey**

Infertility is defined as the inability to get pregnant after trying for one year. In vitro fertilization (IVF) is a type of fertility treatment to help infertile couples get pregnant. IVF involves removing a woman’s eggs from her ovaries and fertilizing them with sperm outside the body. IVF is, generally speaking, the most effective type of fertility treatments and, for some forms of infertility it may be the only treatment option. The average out-of pocket cost of a single IVF cycle ranges from $7,000 to $28,000 (2012). (1) Because patients may need more than one IVF cycle to successfully achieve a pregnancy, the total costs expended may be higher. In one study, the average cost for a couple successfully achieving a live birth was $48,000. (2) In the United States, IVF is not fully covered by private health insurance in most states and otherwise is paid for out of pocket by patients. Public health programs (ex. Medicaid, Medicare) do not cover IVF or any other infertility services.

**Section 1 (Social demographics to be included in survey):**

1. Have you ever had infertility?

☐ Yes

☐ No

2. Do you know anyone who has/had infertility?

☐ Yes

☐ No

3. Do you have any children?

☐ 0

☐ 1

☐ 2

☐ >3

4. Do you plan on having a child/another child?

☐ Yes, in the next year

☐ Yes, in the next 1-5 years

☐ Yes, in the next 10 years

☐ Undecided

☐ No

**Section 2**

5. Do you consider infertility a disease?

☐ Yes, I would consider infertility a disease

☐ No, I wouldn’t consider infertility a disease

6. Do you think private health insurance should cover infertility services, including IVF?

☐ Strongly Agree

☐ Agree

☐ Neither Agree nor Disagree

☐ Disagree

☐ Strongly Disagree

7. If yes, do you think insurance premiums should be increased in order to have IVF coverage for all patients?

☐ Yes

☐ If yes, how much more would you be willing to pay per month?: __________

☐ No

8. Do you think public health programs such as Medicaid should cover infertility services, including IVF?

☐ Strongly Agree

☐ Agree

☐ Neither Agree nor Disagree

☐ Disagree

☐ Strongly Disagree

**Section 3**

*In the following section, we would like your opinion regarding the provision of infertility services to low-income patients.*

9. How do you think infertility impacts low-income in comparison to higher income people?

☐ Infertility has a greater emotional and mental impact on low-income people

☐ Infertility has the same emotional and mental impact on all people, regardless of income

☐ Infertility has less emotional and mental impact on low-income people

10. Doctors should help all people become parents using infertility treatment (including IVF) if they have difficulty achieving pregnancy on their own, regardless of income.

☐ Strongly Agree (proceed to Question 11)

☐ Agree (proceed to Question 11)

☐ Neither Agree nor Disagree

☐ Disagree (proceed to Question 12)

☐ Strongly Disagree (proceed to Question 12)

Skip Question 11 if you answered disagree or strongly disagree for Question 10.

11. If you agree with providing IVF to low-income people, which of the following best describes your reasoning? (Please select all that apply)

☐ Physicians have a social responsibility to provide services, including IVF, to all people regardless of their income status.

☐ Infertility is a disease and treatment should be available and affordable to all people, regardless of income.

☐ All infertile people should have the right to have a family, regardless of income

☐ Lower income people can provide a caring and loving atmosphere for a child.

☐ Other, please specify_____________

Skip Question 12 if you answered agree or strongly agree for Question 10.

12. If you disagree with providing IVF to low-income people, which of the following best describes your reasoning? (Please select all that apply)

☐ IVF is an elective treatment and physicians do not have a social responsibility to provide IVF services to lower income people

☐ Wellbeing of a child born in low-income family may be at risk if he/she grows up impoverished

☐ The pregnancy will likely be supported by federal, state and/or county health programs

☐ There may be a risk of increasing the country’s low-income population and placing strain on natural, financial or social resources

☐ Other, please specify_____________

13. An immigrant couple living illegally in the United States without resident status or citizenship has infertility and requires IVF services to achieve a pregnancy. The resulting child born in the United States will have US citizenship and all accordant rights and benefits.

☐ Doctors should help this couple get pregnant with IVF.

☐ Doctors should not help this couple get pregnant with IVF.

14. For which of the following couples do you think IVF should be available and covered through health insurance? For all of these scenarios, the couples listed have a combined household income of $31,020 (almost 200% above the federal poverty level for a household of 2 people). The average annual expense for raising a child in a two-child married-couple family ranges between $9,330 and $9,980. (3) Select all that apply.

☐ A 30 year-old woman with no previous pregnancies and blocked fallopian tubes, and she and her husband desire a child. IVF is required to achieve a pregnancy.

☐ A 30 year-old woman with three children and a previous tubal ligation (sterilization) recently re-married and desires a child with her current husband. A tubal reversal procedure is not possible, so IVF is required to achieve a pregnancy.

☐ A 30 year-old woman with two children and a tubal ligation performed against her will by a doctor during a cesarean section in her native country desires pregnancy with her husband. A tubal reversal procedure is not possible, so IVF is required to achieve a pregnancy.

☐ A 30 year-old woman with no children was recently diagnosed with breast cancer. If she undergoes chemotherapy soon, her long-term survival rate is excellent. The chemotherapy is likely to affect her ovaries, such that she would be unable to conceive on her own. She and her husband attend an IVF clinic to undergo fertility preservation with embryo freezing to preserve the possibility of having a genetically related child.

References
1. Vitek WS, Galarraga O, Klatsky PC, Robins JC, Carson SA, Blazar AS. Management of the first in vitro fertilization cycle for unexplained infertility: a cost-effectiveness analysis of split in vitro fertilization-intracytoplasmic sperm injection. Fertil Steril 2013; 100:1381-1388.

2. Katz P, Showstack J, Smith JF, Nachtigall RD, Milstein SG, Wing H, Eisenberg ML, Pasch LA, Croughan MS, Adler N. Cost of infertility treatment: results from an 18-month prospective cohort study. Fertil Steril 2011 Mar; 95(3): 915-921.

3. USDA. Expenditures on children by families 2015. Available at: <https://www.cnpp.usda.gov/sites/default/files/crc2015_March2017_0.pdf> Accessed March 27, 2017.
