# Supplementary material for: Public attitudes in the United States toward insurance coverage for in vitro fertilization and the provision of infertility services to lower income patients
Source: F S Rep. 2021 Sep 20;3(2 Suppl):122–9. doi: 10.1016/j.xfre.2021.09.002 (PMC9349243; doi:10.1016/j.xfre.2021.09.002)
Supplement: Supplemental Tables 1-3 [file mmc2.docx]

**TABLES:**

**Table 1. Demographic and socio-economic status (SES) Characteristics Stratified by Support and Nonsupport of IVF Coverage**

| **Demographics** | **Support Insurance coverage (n=568)** | **Do Not Support Insurance coverage (n=189)** | **Neutral (n=270)** | **P-value** |
| --- | --- | --- | --- | --- |
| Age <45 (n=485) | 303 (62.5%) | 69 (14.2%) | 113 (23.3%) |  |
| Age >45 (n=542) | 265 (48.9%) | 120 (22.1%) | 157 (29%) | <0.001 |
| Female (n=535) | 314 (58.7%) | 98 (18.3%) | 123 (22.3%) |  |
| Male (n=492) | 254 (51.6%) | 91 (18.5%) | 147 (29.9%) | 0.03 |
| Not college grad (n=405) | 229 (56.5%) | 59 (39.3%) | 117 (28.9%) |  |
| College grad (n=622) | 339 (54.5%) | 130 (20.9%) | 153 (26.2) | 0.027 |
| Income <$100,000 (n=647) | 370 (57.2%) | 102 (15.8%) | 175 (27%) |  |
| Income >$100,000 (n=380) | 198 (52.1%) | 87 (22.9%) | 95 (25%) | 0.03 |
| Partnered (n=658) | 375 (57%) | 121 (18.4%) | 162 (24.6%) |  |
| Unpartnered (n=369) | 193 (52.3%) | 68 (18.4%) | 108 (29.3%) | 0.24 |
| Race/Ethnicity |  |  |  |  |
| White/European (n=810) | 431 (65.5%) | 160 (19.8%) | 219 (27%) |  |
| Hispanic/Latino (n=59) | 45 (76.3%) | 4 (6.8%) | 10 (16.9%) |  |
| Black/African-American (n=67) | 41 (61.2%) | 8 (11.9%) | 18 (26.9%) |  |
| Asian/Pacific Islander (n=36) | 23 (63.9%) | 4 (11.1%) | 9 (25%) |  |
| Native American (n=8) | 5 (62.5%) | 2 (25%) | 1 (12.5%) |  |
| Other/Multiple (n=47) | 23 (48.9%) | 11 (23.4%) | 13 (27.7%) | 0.06 |
| Religion |  |  |  |  |
| Protestant Christian (n=317) | 160 (50.5%) | 66 (20.8%) | 91 (28.7%) |  |
| Catholic Christian (n=234) | 132 (56.4%) | 43 (18.4%) | 59 (25.2%) |  |
| Jewish (n=27) | 14 (51.9%) | 3 (11.1%) | 10 (37%) |  |
| Muslim (n=19) | 11 (57.9%) | 0 (0%) | 8 (42.1%) |  |
| Buddhist (n=11) | 4 (36.4%) | 0 (0%) | 7 (63.6%) |  |
| Hindu (n=4) | 1 (25%) | 1 (25%)) | 2 (50%) |  |
| Atheist/Agnostic (n=193) | 110 (57%) | 45 (23.3%) | 38 (19.7%) |  |
| Other (n=222) | 136 (61.3%) | 31 (14%) | 55 (24.7) | p=0.006 |
| Insurance Status |  |  |  |  |
| Uninsured (n=56) | 30 (53.6%) | 14 (25%) | 12 (21.4%) |  |
| Private (n=620) | 355 (57.3%) | 113 (18.2%)) | 152 (24.5%) |  |
| Medicare (n=206) | 95 (46.1%) | 46 (22.3%) | 65 (31.6%) |  |
| Medicaid (n=69) | 45 (65.2%) | 4 (5.8%) | 20 (29%) |  |
| Other (n=76) | 43 (56.6%) | 12 (15.8%) | 21 (27.6%) | 0.37 |
| U.S. Region |  |  |  |  |
| Northeast (n=207) | 135 (65.2%) | 25 (12.1%) | 47 (22.7%) |  |
| Midwest (n=233) | 125 (53.6%) | 49 (21%) | 59 (25.3%) |  |
| South (n=317) | 173 (54.6%) | 60 (18.9%) | 84 (26.5%) |  |
| West (n=255) | 124 (48.6%) | 55 (21.6%) | 76 (29.8%) | 0.26 |

**Table 2. Characteristics of Respondents in Respect to Infertility and Personal Reproductive Goals**

| **Reproductive Characteristics** | **Insurance coverage** | **No insurance coverage** | **Neutral** | **P-value** |
| --- | --- | --- | --- | --- |
| No Infertility (n=904) | 496 (54.9%) | 168 (18.6%) | 240 (26.5%) |  |
| History of infertility (n=123) | 72 (58.5%) | 27 (22%) | 30 (24.4%) | 0.75 |
| Does not know someone with infertility (n=461) | 231 (50.1%) | 88 (19.1%) | 142 (30.8%) |  |
| Knows someone with infertility (n=566) | 337 (60.6%) | 101 (17.8%) | 128 (22.6%) | 0.005 |
| Does not desire a child/children (n=822) | 422 (51.3%) | 167 (20.3%) | 233 (28.3%) |  |
| Desire a child/children (n=205) | 146 (71.2%) | 22 (10.7%) | 37 (18%) | <.001 |
| Does not have children (n=440) | 240 (54.5%) | 91 (20.7%) | 109 (24.8%) |  |
| Has a child/children (n=587) | 328 (55.6%) | 98 (16.7%) | 161 (27.4%) | 0.23 |

**Table 3. Attitudes of Respondents on Infertility as a Disease and Societal Obligations regarding Access to Care**

| **Attitudes** | **Insurance coverage** | **No insurance coverage** | **Neutral** | **P-value** |
| --- | --- | --- | --- | --- |
| Infertility is a Disease (n=286) | 222 (77.6%) | 16 (5.6%) | 48 (16.8%) |  |
| Infertility is Not a Disease (n=741) | 346 (46.7%) | 173 (23.3%) | 222 (30%) | <0.001 |
| Doctors Have Social Responsibility (n=217) | 172 (79.3%) | 15 (6.9%) | 30 (13.8%) |  |
| Doctors Have No Social Responsibility (n=810) | 396 (48.9%) | 174 (21.5%) | 240 (29.6%) | <0.001 |
| Immigrants Should Have Access (n=339) | 232 (68.4%) | 29 (8.6%) | 78 (23%) |  |
| Immigrants Should Not Have Access (n=688) | 336 (48.8%) | 160 (23.3%) | 192 (27.9%) | <0.002 |

**Table 4. Multivariable Analysis of Support for Insurance Coverage of Infertility**

| **Variable** |  | **Unadjusted RR** | **Adjusted RR** | ***p*-value** |
| --- | --- | --- | --- | --- |
| **Age** | >45 | Ref | Ref |  |
|  | <45 | 0.5 (0.36-0.71) | 0.63 (0.42-0.94) | 0.03 |
| **Gender** | Female | Ref | Ref |  |
|  | Male | 0.87 | 0.96 (0.67-1.38) | 0.83 |
| **Partner** | No | Ref | Ref |  |
|  | Yes | 1.1 (0.77-1.5) | 1.2 (0.83-1.7) | 0.34 |
| **Education** | Not college grad | Ref | Ref |  |
|  | College grad | 0.67 (0.47-0.95) | 0.62 (0.42-0.91) | 0.01 |
| **Income** | <$100,000 | Ref | Ref |  |
|  | >$100,000 | 0.91 (0.76-1.1) | 0.86 (0.72-1.02) | 0.08 |
| **Know Someone With Infertility** | No | Ref | Ref |  |
|  | Yes | 1.27 (0.9-1.8) | 1.1 (0.8-1.66) | 0.45 |
| **Desire More Children** | No | Ref | Ref |  |
|  | Yes | 1.2 (1.6-4.26) | 1.82 (1.1-3.2) | 0.04 |
| **Believe Infertility is a Disease** | No | Ref | Ref |  |
|  | Yes | 1.93 (1.39-2.5) | 6.8 (3.9-11.8) | <0.001 |
| **Atheist/agnostic** | No | Ref | Ref |  |
|  | Yes | 0.77 (0.52-1.14) | 0.68 (0.44-1.0) | 0.08 |
| **Northeast Region of the U.S.** | No | Ref | Ref |  |
|  | Yes | 2.1 (1.3-3.3) | 2.4 (1.7-3.9) | <0.001 |

**Supplemental Table 1. Reasons for Support and Nonsupport of Insurance Coverage**

| N (%) | Reason for Support (n=568) | N (%) | Reason for Nonsupport (n=189) |
| --- | --- | --- | --- |
| 228 (40.1%) | “People have the right to have a family regardless of income.” | 112 (59.3%) | “IVF is elective & physicians do not have social responsibility to provide it to lower income people.” |
| 217 (38.2%) | “Doctors have a social responsibility to provide infertility services.” | 67 (35.4%) | “The well-being of a child born in a lower income family may be at risk if he/she grows up impoverished.” |
| 167 (29.4%) | “Infertility is a disease and should be available and affordable to all people, regardless of income.” | 70 (37%) | “I am concerned about the risk of increasing the lower income population, which places strain on natural, financial, social resources.” |
| 128 (22.5%) | “Lower income people could provide a caring and loving atmosphere for a child.” | 29 (15.3%) | “I am morally opposed to IVF.” |

**Supplemental Table 2. Participant responses to Scenarios Requiring IVF Coverage**

| Scenario | Support Coverage  N (%)  N=897 |
| --- | --- |
| A 30 year-old woman and her husband desire a child. She has no previous pregnancies and blocked fallopian tubes. IVF is required to achieve a pregnancy. | 731 (79.5%) |
| A 30 year-old woman with three prior children and a previous tubal ligation (sterilization) is recently remarried and desires a child with her current husband. A tubal reversal procedure is not possible, so IVF is required to achieve a pregnancy. | 280 (31.2%) |
| A 30 year-old woman with two children and a tubal ligation performed without her consent by a doctor during a cesarean section in her native country desires pregnancy with her husband. A tubal reversal procedure is not possible, so IVF is required to achieve a pregnancy. | 403 (44.9%) |
| A 30 year-old woman with no children was recently diagnosed with breast cancer. If she undergoes chemotherapy soon, her long-term survival rate is excellent. The chemotherapy is likely to affect her ovaries, such that she would be unable to conceive on her own. She and her husband attend an IVF clinic to undergo fertility preservation with embryo freezing to preserve the possibility of having a genetically related child. | 631 (70.4%) |

**Supplemental Table 3. Demographics of Survey Participants and US Census Data 2020**

| **Survey Demographics**  **(n=1027)** | **Survey Demographics (n=1027)** | **US Census Data 2020 (n=328,239,523)** |
| --- | --- | --- |
| Age <60 (excluding age <18 years old) | 766 (74.5%) |  |
| Age <65 (excluding age <18 years old) |  | 55.2% |
| Female | 535 (52.1%) | 50.8% |
| Male | 492 (47.9%) |  |
| Not college grad | 405 (39.4%) | 67.9% |
| College grad | 622 (60.6%) | 32.1% |
| **Race/Ethnicity** |  |  |
| White/European | 810 (78.9%) | 76.3% |
| Hispanic/Latino | 59 (5.7%) | 18.5% |
| Black/African-American | 67 (6.5%) | 13.4% |
| Asian/Pacific Islander | 36 (3.5%) | 6.1% |
| Native American | 8 (0.7%) | 1.3% |
| Other/Multiple | 47 (4.6%) | 2.8% |
